# Remote Emotion Recognition Using Continuous-Wave Bio-Radar System

**DOI:** 10.3390/s24051420

**Published:** 2024-02-22

**Authors:** Carolina Gouveia, Beatriz Soares, Daniel Albuquerque, Filipa Barros, Sandra C. Soares, Pedro Pinho, José Vieira, Susana Brás

**Affiliations:** 1 Instituto de Engenharia Electrónica e Telemática de Aveiro, Departamento de Electrónica, Telecomunicações e Informática, Intelligent Systems Associate Laboratory, University of Aveiro, 3810-193 Aveiro, Portugal; carolina.gouveia@ua.pt (C.G.); dfa@ua.pt (D.A.); jnvieira@ua.pt (J.V.); susana.bras@ua.pt (S.B.); 2 Colab Almascience, Madan Parque, 2829-516 Caparica, Portugal; 3 Instituto de Telecomunicações, 3810-193 Aveiro, Portugal; ptpinho@ua.pt; 4 Departamento de Electrónica, Telecomunicações e Informática, University of Aveiro, 3810-193 Aveiro, Portugal; 5 Escola Superior de Tecnologia e Gestão de Águeda, University of Aveiro, 3810-193 Aveiro, Portugal; 6 Center for Health Technology and Services Research, Department of Education and Psychology, University of Aveiro, 3810-193 Aveiro, Portugal; fmbarros@ua.pt; 7 William James Center for Research, Department of Education and Psychology, University of Aveiro, 3810-193 Aveiro, Portugal

**Keywords:** micro-Doppler radar, continuous wave, vital signs, bio-radar, emotional recognition, machine learning, classification, pattern recognition

## Abstract

The Bio-Radar is herein presented as a non-contact radar system able to capture vital signs remotely without requiring any physical contact with the subject. In this work, the ability to use the proposed system for emotion recognition is verified by comparing its performance on identifying fear, happiness and a neutral condition, with certified measuring equipment. For this purpose, machine learning algorithms were applied to the respiratory and cardiac signals captured simultaneously by the radar and the referenced contact-based system. Following a multiclass identification strategy, one could conclude that both systems present a comparable performance, where the radar might even outperform under specific conditions. Emotion recognition is possible using a radar system, with an accuracy equal to 99.7% and an F1-score of 99.9%. Thus, we demonstrated that it is perfectly possible to use the Bio-Radar system for this purpose, which is able to be operated remotely, avoiding the subject awareness of being monitored and thus providing more authentic reactions.

## 1. Introduction

The remote assessment of vital signs using radar systems has been highly discussed within the research community, mostly due to its utility and reliability. Such systems, from now on referred as Bio-Radar systems, can capture respiratory and cardiac signals through the measurement of chest-wall displacement during cardiopulmonary function without any contact or body sensor. Generally based in the micro-Doppler effect, these systems transmit electromagnetic waves towards the subject’s chest wall, which are subsequently reflected and received in turn by the radar. The chest-wall displacement changes the traveled path of the electromagnetic waves, which is perceived as a phase modulation on the received signal containing information on the vital signs [1].

The Bio-Radar can be applied in multiple scenarios, either to assist the medical field by providing a non-invasive interaction with the subject, or in other fields/quotidian situations. In this regard, psychology and psychiatry are promising areas of application, considering both clinical and research settings since a more objective assessment and monitoring of the psychophysiological response is not always available or possible to implement. The psychophysiological response may give critical objective information about the emotional response, and there are many scenarios where a non-invasive, effortless, and safe device would be especially critical for this assessment, such as with children [2], or people with sensory hypersensitivity, behavioral problems, and/or difficulties expressing emotions and feelings, (e.g., as observed in people with autism [3]). In these cases, the use of Bio-Radar will allow to study the subject in a more ecological environment since this setup allows a more realistic configuration without intrusiveness in the daily routine.

The ability to assess the subject’s emotions through vital signs has been widely reported and proved to be safe and efficient [4,5]. Emotions trigger the activation of the central and peripheral nervous system, hence being reflected in our physiological response. For instance, the Heart Rate Variability (HRV) measured over the Electrocardiogram (ECG) provides an index of the parasympathetic nervous system (also referred to as vagal tone) [6], which is responsible for allowing our body to regulate and adapt to sudden psychophysiological modifications, required to guarantee homeostasis [7]. Additionally, recent advancements in ECG analysis, such as the COVID-ECG-RSNet model, have demonstrated high accuracy in categorizing heart conditions and related health issues, indicating the potential for further applications in emotion recognition through vital signs [8]. Likewise, there is a wide range of vital signs with informative elements regarding the subjects’ psychological state, some of them being more informative than others. In [9], it was determined that a combination of multiple vital signs can provide better accuracy to identify fear, happiness, and a neutral condition state since all signals contain different information content and patterns that combined may potentially improve the system perceived emotion. The authors also suggest that the ECG is the most effective signal for this scope. Emotions were identified using machine learning algorithms applied to vital signs with the goal to identify patterns. More specifically, in [9], the authors used the Random Forest (RFO) and Artificial Neural Networks (ANN) applied to ECG, Electrodermal Activity (EDA), and Electromyogram (EMG) zygomatic and medial frontal signals. While traditional methods use a chest-band attached to the subject’s chest to acquire the respiratory signal, and the ECG is measured using a three-derivation of skin-contact electrodes, the Bio-Radar system is able to capture both cardiac and respiratory signals concurrently and without requiring direct contact with the subject. This contactless principle can be used in an ecological implementation, providing an unbiased subject’s reactions according to the felt emotions.

Our work started in [10], where we reported some preliminary results identifying the happiness, fear and a neutral condition of nine subjects using only the respiratory signal. In [10], the respiratory signal was acquired simultaneously with the Bio-Radar (bR) and the BIOPAC (from BIOPAC Systems, Inc., United States) (bP), where the latter is a certified measuring equipment serving as a reference for validation. The results obtained [10] for the three emotion identification, demonstrated that the differences between both bR and bP systems are not relevant; however, the bR accuracy might be improved by adding the cardiac signal and by improving the overall setup, extraction algorithms and feature selection.

In order to step forward on this research, our main contribution with the work herein presented is to fully validate the Bio-Radar usage in emotion recognition. The same setup and protocol considered in [10] were followed, but the population under analysis was increased to 20 subjects, and the cardiac signal was included. The happiness, fear and neutral conditions were identified using the respiratory and cardiac signals acquired simultaneously by bR and bP systems. For this purpose, three different machine learning algorithms were applied to the signals acquired by both systems, namely Support Vector Machines (SVM), K-Nearest Neighbor (KNN) and RFO. Furthermore, the features typically used in the literature were surveyed and computed over bR and bP signals to subsequently explore which ones are able to statistically differentiate between emotions.

The paper’s key contributions to emotion recognition using a Bio-Radar system are outlined as follows:

- Demonstrates the radar system’s capability to recognize emotions with a remarkable accuracy of 99.7%, marking a significant advancement in the field of non-contact emotional analysis. It is important to note that these results are specific to the population studied, under the conditions of the study, and using the established emotion induction protocol;
- Establishes that the radar system can match or even exceed the performance of traditional contact-based emotion recognition systems, showcasing its viability as an effective alternative;
- The emotion induction protocol utilized, which has been validated in previous studies [9,11,12,13], effectively mitigated emotional contamination. This approach advanced the successful elicitation of emotions while concurrently eliminating confounding variables. Through this protocol, the study ensured a controlled environment for emotion induction, enhancing the reliability of the emotional responses observed.

This work is divided as the following: Section 2 presents a brief overview about the related work. Section 3 describes the setup used to collect the vital signs and the full experiment conditions. In this section, the signal-processing algorithms applied to recover the vital signs are explained. Section 4 presents the feature extraction process. Section 5 describes the feature selection method. Section 6 presents the classification results, and a comparison with the ones already reported in the literature is presented in Section 7. Finally, Section 8 presents the conclusion.

## 2. Related Work

Emotion recognition using vital signs captured remotely with a radar system has been extensively researched in studies such as [10,14,15,16,17,18,19,20,21,22,23]. However, many of these works lack comprehensive comparisons with certified equipment. In [14], happiness, fear, sadness, and neutral states were identified using a Continuous-Wave (CW) radar operating at 2.4 GHz, combined with an RGB camera, but comparisons were limited to standalone camera or radar results. The study [17] also utilized a 2.4 GHz CW radar for emotion identification but did not incorporate validation studies. Similarly, Ref. [16] distinguished emotions such as happiness, fear, and disgust using an Ultra Wideband (UWB) radar, yet without validation. The work of [15] stands out as the only study that included a validation step, employing respiratory and cardiac signals from a Frequency Modulated Continuous-Wave (FMCW) radar; however, it lacked validation for respiratory signals. Emotion recognition, specifically for emotions like anger, happiness, pleasure, and sadness, was further explored using FMCW radar in [18], yet this study also did not validate both respiratory and cardiac signals. Ref. [19] developed a dual-modal system combining radar and video sensors to improve recognition accuracy under varying lighting conditions. They reported improvements in the results compared to using a single sensor but did not quantify this enhancement or provide precision values for each sensor individually. The work developed in [20] is focused on classifying emotions such as happiness, disgust, and fear by using temporal and spectral features from IR-UWB-based respiration data, but this study did not present measurements with a certified contact sensor for comparison to validate the radar experiments. The authors of the study [23] proposed a unimodal emotion classifier using non-contact ECG signals, achieving high classification accuracies with machine learning classifiers. The authors of the study [22] introduced an effective non-contact emotion recognition system using millimeter wave radar, RF-Emotion, which showed high accuracy in estimating heartbeat intervals and inferring emotional states. Finally, Ref. [21] investigated a deep learning model for emotion recognition, combining radar signals with human face expression images, and demonstrated improved recognition performance. In contrast with previous studies, this one compares FMCW radar measurements with those taken by a contact system, the MI5 smartwatch. Each of these studies contributes unique methodologies and insights, enhancing the capabilities and understanding of remote emotion detection via radar systems.

As one will see later in this work, by comparing the work herein presented with the emotion recognition results reported on [10,14,15,16,17,18,19,20,21,22,23], we could demonstrate that our simple setup and methods are able to improve the results obtained in the literature so far. Furthermore, we also observed that the Bio-Radar system might even outperform traditional contact-based systems considering the same experiment conditions. A proper state-of-the-art discussion and comparison with our work will be conducted further in Section 5.

## 3. Materials and Methods

### 3.1. Setup and Experiment Protocol Description

The Bio-Radar prototype used to acquire the vital signs, operates in CW mode, with a 5.8 GHz carrier frequency to achieve an optimal balance between system sensitivity and resolution. This frequency choice is particularly advantageous for real-world applications due to its simpler hardware requirements and reduced sensitivity to random body motion, crucial for accurate and reliable detection of vital signs. Lower carrier frequencies, such as 5.8 GHz, also offer reduced signal attenuation, which expands the radar’s effective range. This is especially beneficial for concealing the radar within customized objects, enabling a discreet and low-profile system integration. Additionally, operating within the 5.8 GHz Industrial, Scientific, and Medical (ISM) band aligns with our objective of maintaining a fine equilibrium between the sensitivity to physiological signals and the spatial resolution necessary for effective emotion recognition. This strategic selection of the carrier frequency underpins the robust and versatile functionality of our Bio-Radar system in diverse application scenarios.

The setup was composed by a software-defined radio as radio-frequency front-end, namely the USRP B210 board from Ettus Research, United States^TM^, which uses the GNU Radio Companion software (version 3.10.7.0.) The electromagnetic waves were transmitted and received using two 2×2 antenna arrays with circular polarization [24]. This configuration is essential for enhancing the quality of the received signal, particularly the signal-to-noise ratio (SNR). Circular polarizations help in mitigating mutual coupling between antennas, which is crucial for maintaining the integrity and clarity of the signals being captured. In order to avoid the mutual coupling and to improve the path gain [25], the antennas were cross-polarized, with one being right-hand oriented and the other left-hand oriented. Both antennas have an half-power beamwidth of 40° approximately and a gain equal to 11.6 dBi. The setup was operated with a transmitted power equal to 2 dBm at the antenna input.

On the other hand, the bP setup consists on the BIOPAC MP160 Data Acquisition System, which uses the AcqKnowledge 5 software. The respiratory signal (RS) is acquired through the RSP100C module with a transducer chest band attached. The cardiac signal (CS) was obtained through a three-derivation ECG.

**Figure 1:**
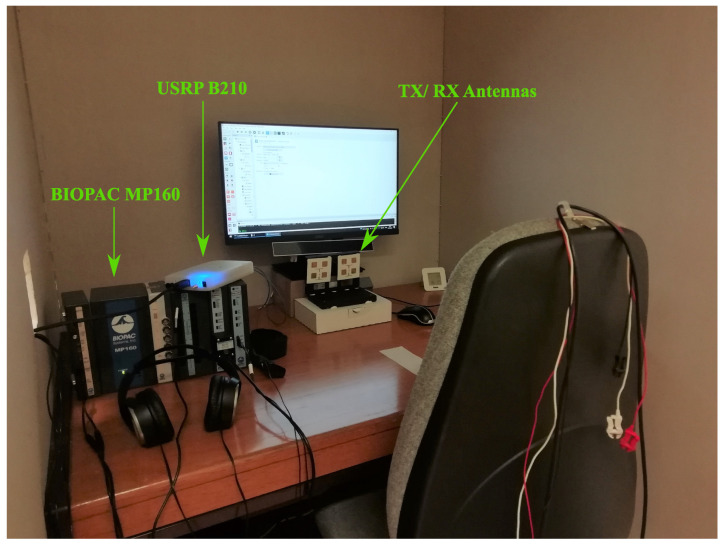
Schematics of the monitoring scenario.

The vital signs of 20 subjects (10 males) within the age range of 20–30 years old were acquired. The study was approved by the Ethics and Deontology Committee of the University of Aveiro, Portugal (No.29-CED/2021). The implemented procedure was in line with the Declaration of Helsinki, and informed consent was obtained from all the participants. The experiment was conducted in three different days, spaced by at least two days. Figure 1 shows the setup disposition and the conditions of the monitoring scenario. The RS and CS were measured using, simultaneously, the bR and bP systems, while the subject was watching videos. The videos were carefully selected based on prior pilot studies, which assessed their effectiveness in eliciting intense emotional responses. This selection was informed by established works, such as in [11,26], providing a validated basis for choosing videos capable of inducing specific emotional states.

To ensure unbiased responses, the subjects were not informed about the content of the videos prior to the experiment. They were only told that their vital signs would be monitored while watching a series of videos. This approach was essential to elicit genuine emotional reactions, free from any preconceived notions or expectations about the video content, thereby maintaining the integrity and accuracy of the Bio-Radar system in emotion recognition. The subject was seated with his/her arms laying on the table in front. This position helped the subjects to remain stable during the experiment. The radar antennas were located at a distance of half a meter in front of the subject (under the computer monitor as depicted in Figure 1).

Each session was composed by a baseline lasting 5 min (where no emotion was induced) and an emotion-inducing period lasting between 25 and 30 min. Each day was dedicated to eliciting a different emotion by showing a set of thematic videos already used in previous studies [9,12]. More specifically, happiness was induced via comedy videos, whereas fear was induced using scary videos, and documentaries were used on the baseline and also to induce the neutral condition. In order to avoid biased results, the subjects did not have prior knowledge of the videos content. Additionally, they were asked not to drink coffee in the previous hour and to avoid attending the session if they were feeling stressed or if an emotionally intense situation had happened.

### 3.2. Vital Signs Extraction

**Figure 2:**
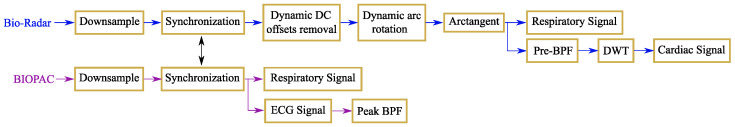
Block diagram of the digital signal-processing algorithm applied to bR and bP signals.

Both bR and bP signals were pre-processed to obtain the RS and CS waveforms. Figure 2 presents a block diagram with the applied algorithms.

Signals from bP were collected at a 1 kHz rate, and the ones from bR were received at a 100 kHz. Thus, the first step in the Digital Signal-Processing (DSP) chain is to downsample both signals, so they were further processed with the same sampling frequency which was 100 Hz. In order to synchronize both bR and bP signals, the subjects were asked to perform a breathing pattern composed by three deep breaths, an apnea period of 10 s and a slow exhale [27]. The considered useful signal starts in the valley (or signal minimum) immediately before the next inhale. Therefore, both signals were subsequently synchronized using this manual method, and then bP and bR signals were processed separately.

Starting with bR signals, the signals received by CW radars are divided in two components, where the useful component corresponds to the reflection occurring in the chest wall, and there is a parasitic component coming from the reflections occurring in the scenario. Furthermore, the front-end used has a quadrature receiver, meaning that baseband signals are complex, and the phase modulation is perceived as an arc in the complex plane. The parasitic component induces DC offsets [28] that alter the position of the arc center. A correct phase demodulation requires that the arc oscillates around the complex origin [28], so these DC offsets must be removed. The prevalent method for DC offset compensation, as described in the literature, is the Park et al. method, which involves circle fitting to determine the center coordinates of the arc, representing the DC offsets [28]. These coordinates are then subtracted from the signal, followed by phase demodulation using the arctangent method to extract the vital signs [29].

However, the Park et al. method has limitations, particularly when dealing with weak signals that lose their arc shape, turning into clusters of dispersed samples. In such scenarios, the fitting process, based on the assumption that all radar samples form a circle, often results in coordinate estimation at the center of the radar samples. This positioning inaccurately pushes the arc to oscillate around the complex origin, leading to erroneous arctangent results.

To address this challenge, the study incorporates an algorithm that utilizes a customized cost function minimization, setting the search area outside the radar samples [24]. This approach ensures that the arc center solution is correctly located outside the radar samples, allowing for proper re-centering of the signal around the origin.

Moreover, the algorithm is dynamic, adjusting over time to accommodate potential DC offsets changes due to body motion [24]. This dynamic application employs an overlapped windowing approach for DC offset estimation. A vector of successive estimations from each window is interpolated, assigning a DC offset coordinate pair to each radar sample. The DC offsets are then smoothly removed by subtracting this interpolated vector from the radar signal.

On the next step, the arc is dynamically rotated to oscillate around the 0° angle. This additional step presents some advantages for the signal processing [24], such as avoiding the wraps that could occur if the signal crosses π. After removing the DC offsets and rotating the arc, the arctangent can be correctly applied to recover the vital signs. At this stage, the extracted signal contains both RS and CS mixed together.

In [27], we compared the performance of several methods dedicated to the CS extraction, and it was concluded that the best approach is composed by a band-pass filter (BPF), used to primarily attenuate the respiratory component (Pre-BPF block on Figure 2), followed by a multi-resolution analysis through the discrete wavelet transform (DWT). The BPF is a 100th order FIR filter, with a pass-band within 0.7–2 Hz, and the DWT coefficients are obtained using the maximal overlap discrete wavelet transform considering 7 decomposition levels [30]. The selected mother wavelet was the Daubechies with 4 vanishing moments and the resultant CS was recovered from the wavelet coefficients using the 5th and 6th decomposition levels [27].

On the bP side, the only signal processing applied after synchronization was dedicated to the ECG signal. A band-pass FIR filter with 15th order and a pass-band equal to 6–20 Hz was applied to highlight the R-peak and remove noise [31].

**Figure 3:**
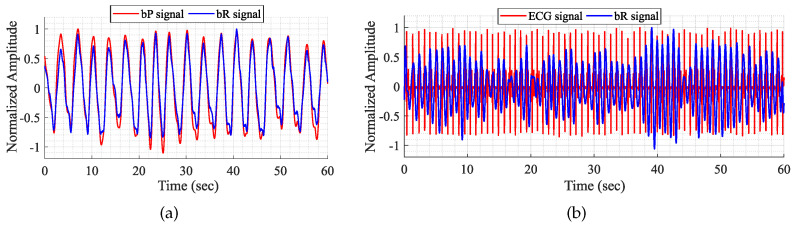
Comparison between the extracted bR signal with the correspondent bP one. (**a**) Respiratory signal; (**b**) cardiac signal.

Figure 3 shows the bR recovered waveforms, superimposed with their correspondent bP signal. For a better comparison, both bP and bR signals were normalized according their maximum amplitude value, and their mean value was removed. Figure 3a shows a high match between the RS of the bR and bP. A high similarity is also observed on the CS of Figure 3b; however, a lack of resolution in the radar CS is also evident since the cardiac peaks position does not always match with the location of the closest R-peak of the ECG, being sometimes frontwards and other times backwards.

The radar follows the same principle as the mechanocardiography (MCG), which measures the mechanical motion induced by the heart at the chest surface [32]. According to [33], MCG signals tend to have interpersonal variations carried by the differences on body mass index, age, sex, among other health-related factors, resulting in different beat morphologies. Furthermore, Ref. [33] also highlights that it is challenging to obtain accurate interbeat intervals (IBIs) under these circumstances, leading to inaccurate HRV parameters. The HRV provides an index of the vagal tone by representing the change in the time interval between successive cardiac peaks, or in other words, the IBI variability. The following subsection describes the approach conducted to still take advantage of the HRV information, despite the inaccurate IBI assessment.

### 3.3. Window-Based HRV Parameters

The HRV analysis can be performed in the time and frequency domains, but only some specific parameters can provide direct information regarding the vagal tone. The time domain parameters encompass, for instance, the standard deviation of all IBI (SDNN), the root mean square of the successive differences of the IBI (RMSSD), or the percentage of successive normal sinus IBI taking more than 50 ms (pNN50). SDNN represents all the cyclic components that induce the variability, and the vagal tone is mostly represented by RMSSD and pNN50 [6]. On the other hand, the frequency domain analysis is performed by analyzing the absolute power in specific frequency bands. In this case, the vagal tone is represented by the high-frequency (HF) band, specifically defined within 0.15–0.4 Hz. However, this parameter is also influenced by the respiration since the heart rate varies with the respiratory cycle. In turn, the RMSSD is not affected by the respiratory signal.

Considering the ability to minimize the error and leverage the pertinent information these parameters contain, only the time domain HRV parameters were considered in this work. In the study, Heart Rate Variability (HRV) analysis plays a pivotal role, particularly in the context of emotion recognition. Given the specific characteristics of radar signals at 5.8 GHz, including their relatively lower resolution as shown in previous research [34]. These parameters are known for their susceptibility to signal resolution. This sensitivity is notably more evident with frequency domain parameters, which are further influenced by factors such as the choice of FFT window and the spectral impact of respiratory activity.

Time domain HRV parameters offer a more direct calculation method, devoid of intermediate steps that might introduce additional variables or errors, such as those encountered in FFT-based computations. For example, the RMSSD (Root Mean Square of Successive Differences), a time domain parameter, effectively conveys information about parasympathetic activation. This rationale supports the decision to use time domain parameters, as they provide a more synthesized assessment without the complexity and potential inaccuracies of frequency domain parameters.

Moreover, the study employed a sliding window approach for HRV computation, addressing the resolution limitations of the radar signal. This approach was critical for enhancing the accuracy of HRV analysis. By comparing the traditional methods with the window method, it was found that the latter substantially minimizes errors in HRV parameter estimation. Such an approach ensures more precise and reliable HRV analysis, essential for accurately assessing emotional states through HRV metrics. This decision aligns with the overall aim of the study to develop a robust and reliable system for emotion recognition using non-contact radar technology.

**Figure 4:**
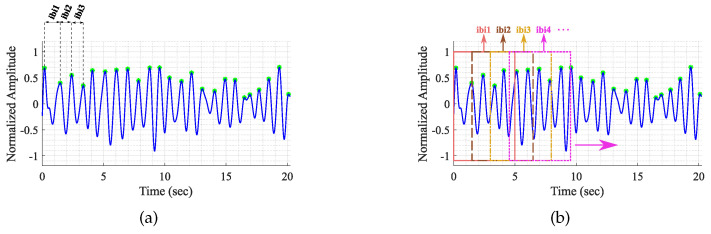
Illustration of the IBI computation. (**a**) Through conventional method; (**b**) Through sliding window.

As mentioned previously, the HRV parameters are obtained through the analysis of the differences between consecutive peaks as depicted in Figure 4a. Due to the high variability of the radar cardiac peaks location, using these IBI values generates a high error in the HRV parameters computation. The sliding window approach depicted in Figure 4b can reduce this error substantially. This approach consists of computing the IBIs inside a window of 5 s (the 5-s window duration was selected to guarantee at least 5 peaks and hence 4 IBIs, considering as reference the 60 BPM of heart rate) and compute its median value. The median was used to neglect outliers. The window moves forward only enough to overlap the previous one 75%, and a new median value is computed. In the end, the IBI vector is the result of these median values, rather than the difference of consecutive peaks.

**Figure 5:**
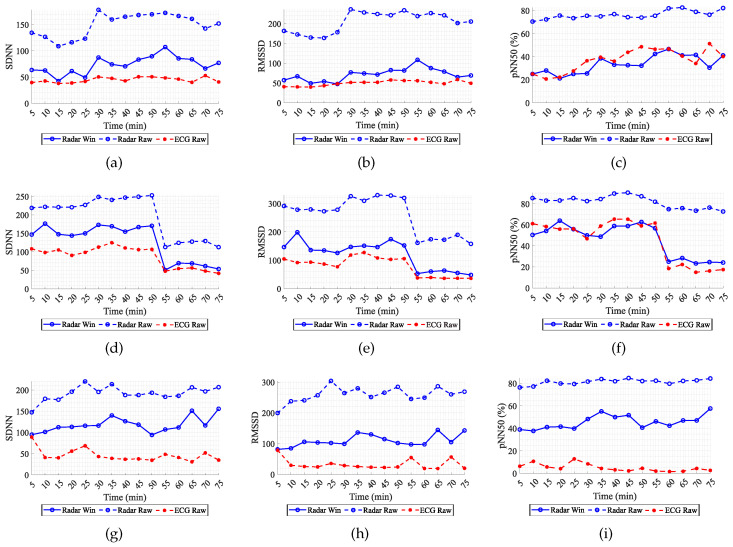
Comparison of the time domain HRV parameters computed using the conventional method on radar signal, the sliding window method and the original ECG result. (**a**) SDNN for ID01; (**b**) RMSSD for ID01; (**c**) pNN50 for ID01; (**d**) SDNN for ID06; (**e**) RMSSD for ID06; (**f**) pNN50 for ID06; (**g**) SDNN for ID10; (**h**) RMSSD for ID10; (**i**) pNN50 for ID10.

Figure 5 shows the obtained results for three different subjects—ID01, ID06 and ID10—for all emotional conditions. These graphs show the variation of the HRV parameters over time, computed every 5 min using the ECG signal; the radar signal directly (let us refer to this as the conventional method); and after applying the sliding window method over the radar signal. First of all, it is clear to see the discrepancy between the ECG and the conventional method applied to the radar. In average, the SDNN can vary between 105 and 145 ms^2^, the RMSSD 155–227 ms^2^, and the pNN50 40–75%. By applying the sliding window, this error decrease to the ranges of 30–72 ms^2^, 22–77 ms^2^ and 6–40% for the SDNN, RMSSD, and pNN50, respectively, for these subjects. More specifically, for subject ID01, the mean value of the sliding window method approximates highly the mean value obtained for the ECG, and for subject ID06, the sliding window results keeps track of the tendency of the ECG. On the other hand, subject ID10 still presents a considerable error in relation to the ECG, but it is decreased considerably in relation to the conventional method applied to the full radar. In sum, the median values of small size windows may remove outliers and still preserve useful information regarding the vagal tone of the subject.

## 4. Features Extraction

After processing the bR and bP signals, they were all divided into one-minute segments, leading to a balanced dataset with a size equal to 1626 min (542 min per emotion).

Both the bR and bP systems were employed for feature selection, with the aim of distinguishing between different emotional states. The process of selecting specific features for these systems was crucial, given the distinct methodologies and capabilities of each system.

For the bP system, the cardiac signal is measured using an electrocardiogram (ECG), which is an electrical signal with high resolution and a specific waveform. On the other hand, the Bio-Radar system captures the heart signal from the movement of the chest. The cardiac signal from the bR corresponds to the ’R’ peak in the ECG [35]. This difference in the measurement approach led to the computation of Feature 11 (F11) differently for each system.

In addition to the cardiac signal, the Bio-Radar has the unique ability to capture the subject’s movement, which is not feasible with the bP system. This capability of the Bio-Radar adds an additional dimension to the emotion recognition process.

Given these differences, a statistical study was conducted, which resulted in the selection of distinct features for each system. The selected features for each system were tailored to maximize the information content relevant to their respective methodologies. Despite the variations in feature selection, both systems were effectively utilized to differentiate between emotions, leveraging their respective strengths.

Several features were computed over them to further build the observation matrix which will be used by the classifiers. In order to assure repeatability, the code developed for features extraction, statistical analysis and classifiers implementation is available at https://github.com/ctsgouveia/RadarEmotions.

For starters, the works [9,14,15,16,17] were analyzed and almost all features were considered and extracted from one-minute segments. Thereafter, a statistical study was conducted over all features, aiming to select the ones that provide significant information to differentiate between the different emotions. The features considered at this stage can be divided into four different categories—waveform features, statistical features, spectral features and HRV features—and they are compiled in Table 1. The same features were computed for both bR and bP signals, excepting one punctual exception detailed later.

Waveform, statistical and spectral features were computed directly over one-minute segments, and more details are now provided: features F5–F10 correspond to the mean absolute value of the first and second derivatives of CS, RS, normalized RS and the IBI of the CS; F13 is the mean value of the width of peaks and valleys; F39–F44 correspond to the PSD over separated frequency bands, namely, 0–0.1 Hz, 0.1–0.2 Hz, 0.2–0.3 Hz, 0.3–0.4 Hz, 0.4–0.9 Hz and 0.9–1.5 Hz; F45 is the PSD ratio between a lower-frequency range within 0.1–0.4 Hz and a high-frequency range within 0.5–1.5 Hz; and F11 represents the energy ratio between RS and CS, and it is computed differently for bR and bP signals. For bR, F11 corresponds to the ratio between the RS and the signal resulting from the Pre-BPF application (mentioned in Section 2). This filtered signal was considered instead of the wavelets results for containing all high-frequency spectral components. For bP, the F11 is the ratio between the signal coming from the chest-band and the ECG signal.

**Figure 6:**
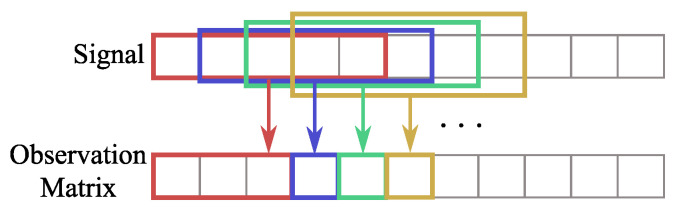
Illustration of how HRV features are computed and assigned to each observation.

On the other hand, the HRV features needed to follow a different computation strategy. The observation matrix applied to all classifiers has a N×M dimension, where *N* is the number of features and *M* is the number of observations, and each observation corresponds to the feature result computed over a one-minute signal. However, the Task Force in [36] recommended to use signals with at least a 5 min duration to compute the HRV parameters, as a gold standard. Therefore, we computed the HRV parameters using sliding windows with a 5 min length, moving forward at a 1 min pace as depicted in Figure 6. The observation number corresponds to the central minute of the 5 min window.

More details can be also provided about the HRV parameters: F47 and F49 are related to the respiratory peaks, so they were computed in one-minute segments; for F46–F49, the results are presented in log transform to adjust for the unequal variance since the data presented a non-normal distribution [6]; F51–F52 were computed using a quadratic fitting and considering a short range ($\alpha_{1}$) between 20 and 60 beats and a long range ($\alpha_{2}$) between 10 and 100 beats [37]; and F53–F60 correspond to the Poincaré plot features, namely, the standard deviation in crosswise (SD1), the standard deviation in lengthwise (SD2), the ratio between SD1 and SD2 (SD12) and the square root of the IBI variance (SDRR). All the Poincaré plot parameters were computed without and with delay (i.e., m=1 and m=10, respectively). Furthermore, another procedure was conducted over the HRV features computation.

Features were computed over the signals corresponding to the baselines and the induced conditions. In order to guarantee a user-independent and day-independent analysis, the mean value of the baseline features on a given day and for a specific subject was subtracted from the features computed for the emotional condition of that day [38].

## 5. Feature Selection

**Figure 7:**
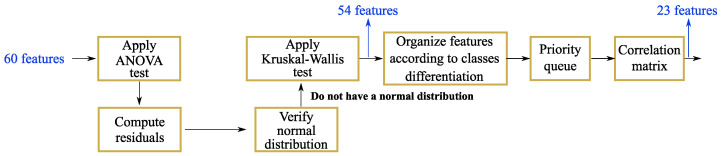
Workflow of the statistical study for features selection.

After computing all features and normalizing them, the most informative ones were selected through a statistical study. Figure 7 depicts the workflow being followed, showing as an example the features selected for the bR case.

First of all, the one-way analysis of variance (ANOVA) test was applied over all features in order to inspect if the resultant residuals have a normal distribution. In this case, it was considered the most voted answer of three different tests: the Kolmogorov–Smirnov test, the Anderson–Darling test and the Lilliefors test. All features presented a non-normal distribution. Secondly, an hypothesis testing was performed to verify which features represent statistical differences between the classes being identified. Since all features have a non-normal distribution, the Kruskal–Wallis test was conducted since it makes no normality assumption about the population distribution [39]. The Kruskal–Wallis test analyzes the variance of ranks, in this case, by inspecting the medians of the features. At this stage, one intends to find the features presenting different medians for distinctive classes. For each feature, the resultant *p*-value was inspected, and only the features with p<0.05 were considered further. Subsequently, the Pairwise T test with one-step Bonferroni correction was applied to analyze which pair of classes each feature can differentiate.

The third step of this analysis is building a priority queue of features. For each binary classification, namely, fear vs. happy (FH), fear vs. neutral (FN), and happy vs. neutral (HN), features were settled in a column, in ascending order according to the *p*-values obtained in the Pairwise T test. Thus, the first row of each column presents the features that better distinguishes the correspondent classes (with lower *p*-values) and the last row presents the less significant features (with higher *p*-values). From this point, the queue is built over the rows, prioritizing the elements with lower *p*-values, and removing the repeated entries.

**Figure 8:**
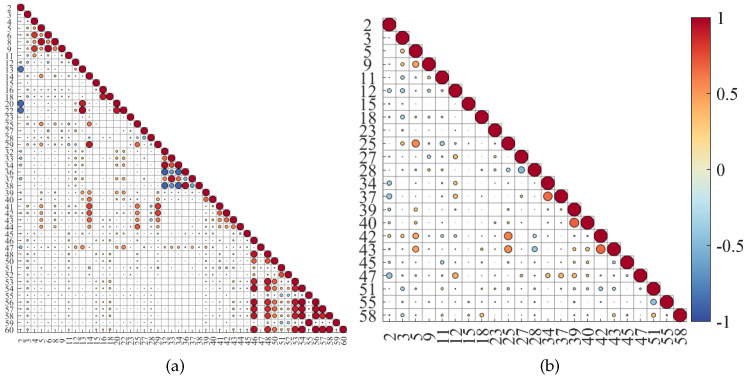
Correlatio n matrix for feature selection for bR: (**a**) After the Pairwise T test, (**b**) after removing redundant features.

The final step of this study is creating a correlation matrix in order to remove features with redundant information and simplify the classification models. If a given pair of features presents high correlation, it indicates that the variation of one affects directly the other. In this case, one of them can be removed keeping always the one with more priority in the queue [40]. Thus, the final selected features were the ones presenting correlation values inferior to 0.7, selected according to their position in the priority queue. As an example, Figure 8 shows the correlation matrices before and after removing the redundant elements for bR signals.

At the end, one could reduce an observation matrix with 60 features to 23 features for the bR and 19 features for the bP. Table 2 presents the list of features for both systems, in decreasing order of importance, according to their priority queues.

### Feature Selection Discussion

The features presented in Table 2 are the ones that are able to distinguish the different classes. In total, there are 11 features commonly selected by both systems, namely, F25, F55, F51, F34, F2, F45, F27, F3, F12, F43, and F9, which is approximately half of the set. This fact could indicate that both systems present a similar content of information, despite the different particularities related to the signals nature. The body motion is highly accounted on the bR case since the first four features with more priority are dedicated to RS (and, therefore, indirectly related with RBM), while the first four ones on the bP case are relative to the CS. In total, five and eight features were selected from the spectral and statistical categories on the bR case, whereas only three and five features were selected for the bP case for the same categories.

One should note that the same features were computed over the bR and bP signals for a fair comparison. This work aims to verify if both systems have the same ability to detect emotions in the same conditions, rather than choosing the one with the best performance. Therefore, it should be stated that more appropriate features can be selected for the bP system, such as the frequency domain parameters of the HRV, and in that case, the features selected for the bP might be different, as well as the results obtained further.

## 6. Classification Results

After selecting which are the features with pertinent information to differentiate between classes, three machine learning algorithms were implemented—SVM, KNN and RFO. The classifier hyperparameters used were the same previously as those presented in [10], and the considered evaluation metrics were the following:

- Cross-validation (CV) using the leave-one-out strategy [10];
- Testing stage ($T_{30}$) using a hold-out strategy where 70% of the dataset of each condition was used to train the model and the remaining 30% was used to test it. The partition of data was performed randomly and repeated 20 times. The results are presented as the mean value and standard deviation of the accuracy and F1-score.

The following subsections present the performance results of the classification accuracy, considering two different problems. The first is a binary one, considering the testing cases FH, FN and HN. Then, the multiclass problem is dedicated to test the HNF condition.

### 6.1. Binary Problem

The results obtained for the binary problem are presented in Table 3. The first aspect that should be highlighted is that the accuracy increased in comparison to our previous work presented in [10]. By comparing only the CV results (which was the performance metric used in [10]), one can conclude that by increasing the set of features, removing the individual and daily dependencies, and by carefully selecting the most important ones, the performance is increased for both bR and bP systems. The impact is more prominent in the bR system, which increased on average 19.4%, 21.9%, and 19.8% for the HN, FH, and FN conditions, respectively. An inferior rise was observed for the bP, being 8.3%, 15.3%, and 7.5% for the same emotional conditions. Other important aspect is that, while in [10] the FN condition was the one that obtained the best results, in this case, no condition stood out, presenting all balanced performance results. This effect was observed for both the bR and bP cases.

Observing now the test performance using the remaining 30% of the data not used for training ($T_{30}$), the model robustness was validated since the testing results do not differ largely from the CV ones. This was observed for all classifiers, whereas each presented similar but yet different performance results. For the bR case, the SVM classifier presented a mean performance of 84.5% for all binary cases, while the KNN and RFO performed better with an average of 97.5% and 99.5%, respectively. In general, the bR case presented better accuracy than the bP case for both CV and $T_{30}$, where the difference did not exceed ≈8%.

One additional test was carried out. In $T_{30}$, only 19 subjects were considered in 70:30 data partition, leaving one subject out to serve as new data. In this case, the classifiers performance was significantly worse, and the standard deviation increased abruptly, indicating a large variance in results. This was somehow expected since the subject used in the test was not considered in the training phase. If the subject being tested separately does not have similarities with the data being used to train the model, the model will not fit with their behavior, and the accuracy will decrease. Hence, the data of 20 subjects may not be enough to generalize the models.

The differences observed for bR and bP systems can be justified with the occurrence of random body motions, which are easily captured by bR but are less evident in bP signals. Each emotional condition can provoke motions specific to the emotion and emotional intensity experienced by the individuals [13]. For instance, the fear induces fright and comedy videos lead to laughs. Since bR is more affected by external motions and the bP results are exclusively dedicated to patterns in vital signs, the motion patterns might be seen as an additional information element.

### 6.2. Multiclass Problem

Table 4 presents the results of the multiclass problem. For this problem, other metrics were also evaluated, namely, the F1-score since it might provide a fairer comparison for this specific case. This metric establishes a balance between recall and precision, and it is more appropriate for short datasets applied in a multiclass problem [9,16].

Starting with the results obtained for the multiclass problem using all the features selected in Section 3, similar performance results were achieved in comparison with the binary problem. The CV is again close to the $T_{30}$, indicating the robustness of the models. Better results were obtained for the bR on the $T_{30}$, which could differentiate the three emotions with 99.7% accuracy and 99.9% F1-score using the RFO classifier, while the bP presented an accuracy almost 10% below, with 87.8% and 92.7% F1-score, also using the same classifier. Since in the binary problem approximately the same performance was verified for all cases and it was around 99% for the bR case, these multiclass results were expected. As regards the remaining classifiers, for both bR and bP cases, the SVM classifier was the worst, and KNN presented a performance similar to that of the RFO classifier.

## 7. Discussion of the State-of-the-Art Related Works

As previously mentioned, emotion recognition using vital signs captured by radar systems is a topic lacking development. To the best of our knowledge, only four works [14,15,16,19,21] used radars for the same purpose, and the obtained results are in line to the ones herein presented. Table 5 sums up the characteristics and the best results obtained for each work. The results are exclusively for the multiclass strategy (i.e., the ability to distinguish all emotions) and for a person-independent scenario, where the dataset is relative to all subjects.

All works used different radar front-ends, so it is not possible to compare the performance of all works directly. For instance, similarly to our work, Refs. [14,17] used also CW radars but operating with a lower carrier frequency. The carrier frequency influences the sensitivity of the system to detect micro-motions such as the ones related with the heart motion at the chest surface [41]. In this sense, higher carrier frequencies are more sensitive and might be able to detect more easily the micro-motions caused by the heart. Nonetheless, such a level of sensitivity make ssignals prone to being highly affected by random body motion during the monitoring period, and high carrier signals are also severely attenuated. The works [15,16] used higher carriers but with different radar operation modes, namely, FMCW and UWB, which have different implementation challenges in comparison with CW radars.

In general, all datasets are short, varying between 5 and 35 subjects. In contrast, the current work was dedicated to 20 subjects, which is enough to detect large effects of the HRV parameters according to [42], hence providing more robust results. Regarding the used vital signs, the works [14,15] are in line with our work, using both RS and CS to compute features, while [16,17] only considered the RS. The features normalization using the baseline information was only implemented in [14,15].

The emotions under study are different among all the considered studies. In our work, we are only dedicated to three emotions, which are the most focused ones in these references, being happiness, neutral condition and fear. Other studies also include sadness, anger, pleasure and disgust. The works [14,15,17] presented an accuracy varying between 67 and 72% for detecting four emotions, where [14,17] were dedicated to the same emotions, and [15] was focused on a different set. On the other hand, our work and [16] were dedicated to three emotions and although we obtained a better accuracy of 99%, Ref. [16] was not focused on the same emotional set and used a completely different radar setup.

It is also important to mention that the process of emotion elicitation, as well as the general methodological procedure, may have played a role in the results discrepancy. For instance, depending on the procedure used to elicit emotions and whether or not this elicitation was successful, it may be more difficult to distinguish between different emotional conditions [43,44]. Also, more authentic reactions may be obtained if the subject is not aware of the video’s content, and if the emotional inductions are sufficiently spaced in time (which also helps do decrease emotional contagion from one session to another). The works [14,15,17] induced all the emotions on the same day and [16] on consecutive days. We followed the same induction protocol used in [9], inducing emotions on different and spaced days, to try to avoid emotional contagion as much as possible [12]. In future studies, it would be interesting to explore the ability of the bR to distinguish different emotions elicited in more ecologically valid settings, i.e., framed in a real-life context. Although inducing emotions in the laboratory allows to control for important confounding variables, this procedure may keep the emotional responses away from the authenticity and variability found in daily life [12,43,45].

In addition to the works presented in Table 5, the work of Pinto et al. [9] can also be included in this discussion since they conducted a study on emotion recognition focused on the same emotional set and following the same emotion induction protocol. Their dataset was composed by the ECG, EMG, and EDA of 55 subjects, acquired with BIOPAC. They also conducted a classifier performance analysis similar to the one herein presented, where one can stand out their (c) case [9]. In the (c) case, 30% of each emotional condition of the test data was included in the training process, which is similar to our $T_{30}$, but we used 70% for training instead of only 30%. Comparing now the F1-score results, in [9], they obtained 73% using the RFO classifier, and we were able to obtain 99.9%. Our results are thus in line with the ones obtained in [9] and the observed differences might be mostly justified with the sample size, the number of observations used for training and testing, and the different vital signs used in both works.

## 8. Conclusions

In this work, the Bio-Radar system is presented as a possible and reliable tool to assist in emotion recognition. Its remote operation allows to assess the unbiased and authentic reactions of subjects in an emotional induction scenario.

A validation was carried out by comparing the Bio-Radar classification results with the ones obtained using the BIOPAC as a reference. The work is a follow up of a pilot study presented in [10] which used only the RS signal to distinguish the same set of emotions. Therefore, in this case, the CS was included in the analysis, a total of 60 features were computed, and a statistical study was implemented to identify the most informative ones. The results accuracy increased more than 20% in comparison to our previous study.

In general, the obtained results demonstrated that if classifiers include the population under study in the training process, the bR outperforms the bP in the emotion recognition, considering the same set of 60 features as a starting point. More specifically, according to the F1-score obtained in the multiclass problem, we were able to recognize ≈100% of the cases.

The statistical study implemented for both systems provided a reduced set of features, where ≈50% was common in bR and bP, indicating that signals acquired by radar contain equivalent information. Additionally, the body motion captured by bR could possibly explain its superior performance in comparison with the bP. As already mentioned in [10], frights have an inherent motion pattern as do chuckles, which are captured more easily by the bR than the chest-band of the bP.

Finally, the literature was also analyzed, and considering that a direct comparison cannot be performed fairly, one can only conclude that the results obtained in our work are in line with the ones presented in the countable works in the literature.

## Figures and Tables

**Table 1:** Initial set of features.

Category	Feature No.	Description	Applied Signal
Waveform	F1–F2	Signal Rate	CS and RS
	F3–F4	AppEn	CS and RS
	F5–F7	First derivative	RS, RS-N, IBI-CS
	F8–F10	Second derivative	RS, RS-N, IBI-CS
	F11	Energy Ratio	RS
	F12	Kurtosis	RS
	F13	Peak Width	RS
	F14	Variance	RS
Statistical	F15–F18	Sk, Med, IQR, Av	IBI-CS
	F19–F22		IBI-RS
	F23–F26		CS
	F27–F30		RS
	F31–F34		Inhale (RS)
	F35–F38		Exhale (RS)
Spectral	F39–F44	PSD	RS
	F45	PSD ratio	RS
HRV parameters	F46–F47	SDNN	IBI-CS, IBI-RS
	F48–F49	RMSSD	IBI-CS, IBI-RS
	F50	pNN50	IBI-CS
	F51–F52	DFA2 $\alpha_{1}$ and $\alpha_{2}$	IBI-CS
	F53–F56	Poincaré plot for m=1	IBI-CS
	F57–F60	Poincaré plot for m=10	IBI-CS

CS—cardiac signal; RS—respiratory signal; RS-N—normalized respiratory signal; IBI-CS—interbeat Interval for CS; IBI-RS—interbeat interval for RS; AppEn—approximate entropy; Sk—skewness; Med—median; IQR—inter-quartile range; Av—average; PSD—power spectral density; SDNN—standard deviation of all IBI; RMSSD—root mean square of the successive differences of the IBI; pNN50—percentage of successive normal sinus IBI taking more than 50 ms; DFA—detrended fluctuation analysis.

**Table 2:** List of selected features for bR and bP systems.

Bio-Radar		BIOPAC	
Feature	Description	Feature	Description
F28	Median of RS	F25	IQR of CS
F34	Mean of inhale time	F55	SD12 for m=1
F2	RS rate	F24	Median of CS
F43	PSD in 0.4–0.9 Hz	F51	DFA2 $\alpha_{1}$
F23	Skewness of CS	F34	Median of inhale time
F3	AppEn of CS	F2	RS rate
F42	PSD in 0.3–0.4 Hz band	F45	PSD ratio
F9	Second derivative of RS-N	F27	Skewness of RS
F27	Skewness of RS	F41	PSD in 0.2–0.3 Hz
F25	IQR of CS	F3	AppEn of CS
F58	SD2 for m=10	F10	Second derivative of IBI-CS
F51	DFA2 $\alpha_{1}$	F4	AppEn of RS
F37	IQR of exhale time	F12	Kurtosis of RS
F40	PSD in 0.1–0.2 Hz band	F35	Skewness of exhale time
F18	Mean of IBI-CS	F54	SD2 for m=1
F45	PSD ratio	F43	PSD in 0.4–0.9 Hz
F39	PSD in 0–0.1 Hz band	F19	Skewness of IBI-RS
F5	First derivative of RS	F9	Second derivative of RS-N
F55	SD12 for m=1	F48	RMSSD for IBI-CS
F11	Energy ratio		
F12	Kurtosis of RS		
F15	Skewness of IBI-CS		
F47	SDNN of IBI-RS		

**Table 3:** Accuracy results for the binary problem.

Accuracy (%)	HN [m±std]		FH [m±std]		FN [m±std]		
	CV	$T_{30}$	CV	$T_{30}$	CV	$T_{30}$	
SVM	82.2±3.1	83.6±4.7	83.6±2.3	83.7±3.9	84.9±2.3	86.1±3.1	bR
	80.1±1.5	80.3±3.1	81.7±2.5	83.7±2.5	79.8±1.3	79.6±2.7	bP
KNN	97.2±0.5	97.3±0.7	97.9±0.4	97.5±0.9	97.4±0.4	97.8±0.8	bR
	91.8±0.8	91.8±1.4	91.8±0.8	91.8±1.4	91.4±0.7	91.1±2.3	bP
RFO	99.5±0.2	99.6±0.4	99.2±0.4	99.4±0.5	99.3±0.3	99.6±0.7	bR
	91.0±1.0	91.9±1.6	90.2±1.3	91.6±2.2	91.2±0.7	91.2±1.6	bP

**Table 4:** Performance results for the multiclass problem.

	Accuracy [m±std]		F1-Score [m±std]	
	CV	$T_{30}$	$T_{30}$	
SVM	76.8±2.1	76.7±3.5	85.2±3.9	bR
	72.9±2.3	74.7±2.5	94.0±4.7	bP
KNN	96.0±0.4	96.4±0.8	98.0±1.0	bR
	86.2±1.0	86.6±1.3	92.8±2.0	bP
RFO	99.2±0.3	99.7±0.3	99.9±0.2	bR
	86.5±1.0	87.8±1.4	92.7±1.9	bP

**Table 5:** Comparison of the results obtained in different works on emotion recognition using radar systems.

Work References		[14]	[15]	[16]	[21]	[19]	Current Study
Setup		CW radar @ 2.4 GHz + RGB camera	FMCW radar @ 5.46–7.25 GHz	UWB radar @ 7.29–8.79 GHz	FMCW radar @ 76–81 GHz	CW radar @ 2.4 GHz + camera	CW @ 5.8 GHz
Vital Signs		RS and CS	RS and CS	RR	RS and CS	CS	RS and CS
N° Observations		2010 (1 min) of 18 Sub.	400 (2 min) of 11 Sub.	315 (5 min) of 35 Sub.	1200 (1 min) of 20 Sub.	512 (1 min) of 10 Sub.	1626 (1 min) of 20 Sub.
Tested Classifiers		RFO	SVM	KNN, ETC, ADB, GBM, SV, HV, CNN, MLP	ER-CNN, 1D-CNN, Bi-LSTM	CNN	SVM, KNN, RFO
Emotions		H, N, F, S	H, S, A, P	H, F, D	H, N, S, A	H, S, N, F	H, N, F
N° Features		63 → 23	27	3 → 1	-	-	60 → 23
Performance Evaluation	CV	10-fold	-	10-fold	-		Leave-one-out
	Test	Hold-out with 70:30 ratio over the dataset	Hold-out with 11/12 and 1/12 for test	Hold-out with various ratios over the dataset	Hold-out with 80:10 ratio over the dataset and 10 ratio for validation	Hold-out with 80:10 ratio over the dataset and 10 ratio for validation	Hold-out with 70:30 ratio over 19/20 and 1/20 for test
Results	CV	89.6%	-	66%	-	-	99.2%
	Test (Accuracy)	71%	72.3%	76% with 80:20 ratio	91%	90.8	99.7% with 70:30 ratio

CW—continuous wave; FMCW—frequency-modulated continuous wave; UWB—ultra wideband; CS—cardiac signal; RS—respiratory signal; RR—respiratory rate; RFO—random forest; SVM—support vector machines; KNN—K-nearest neighbor; ETC—extra tree classifier; DB—AdaBoost classifier; GBM—gradient boost machine; SV—soft voting; HV—hard voting; CNN—convolutional neural networks; MLP—multi-layered perceptron; Sub.—subjects; H—happiness; N—neutral condition; F—fear; S—sadness; A—anger; P—pleasure; D—disgust; CV—cross-validation.

## Data Availability

None of the experiments was preregistered. The code developed for the features extraction, the statistical analysis and the classifiers implementation is available at https://github.com/ctsgouveia/RadarEmotions (accessed on 20 August 2023). A dataset of one subject is available as example to run the code. The developed code is available at https://github.com/ctsgouveia/RadarEmotions.
